# Comparison of growth patterns in healthy dogs and dogs in abnormal body condition using growth standards

**DOI:** 10.1371/journal.pone.0238521

**Published:** 2020-09-23

**Authors:** Carina Salt, Penelope J. Morris, Richard F. Butterwick, Elizabeth M. Lund, Tim J. Cole, Alexander J. German

**Affiliations:** 1 Waltham Petcare Science Institute, Mars Petcare, Waltham on the Wolds, Leicestershire, United Kingdom; 2 Banfield Pet Hospital, Vancouver, WA, United States of America; 3 Great Ormond Street Institute of Child Health, University College London, London, United Kingdom; 4 Institute of Life Course and Medical Sciences, University of Liverpool, Neston, Cheshire, United Kingdom; University of Illinois, UNITED STATES

## Abstract

In dogs, optimal growth is critical for future health and wellbeing. Recently, a series of evidence-based growth standards, based on bodyweight, were developed for male and female dogs across 5 different size categories. The aim of the current study was to compare growth curves depicted by the standards with patterns of growth in dogs that were either healthy, had abnormal body condition, or had various diseases with the potential to affect growth. The data came from 2 research colonies in Europe (France and UK), and a large corporate network of primary care veterinary hospitals across the USA. Age and bodyweight data were used to model growth in healthy dogs, in dogs that became overweight or underweight by 3 years of age, and in dogs with diseases associated with altered growth. Centile line crossing during the growth phase was uncommon in healthy dogs, with <5% of dogs crossing >2 centile lines. In contrast, centile line crossing was more frequent in dogs with abnormal growth patterns or abnormal body condition. Dogs that developed obesity by 3 years grew faster than the growth standards predicted, and 68% crossed ≥2 centile lines in an upwards direction. Dogs with conditions associated with accelerated growth also grew faster than expected, and 54% crossed ≥2 centile lines. In contrast dogs that became underweight by 3 years gained weight slower than expected, and 49% crossed ≥2 centile lines in a downwards direction. These results suggest that the growth standards are useful for monitoring healthy growth in dogs. Prospective studies are now required to confirm these findings and to determine whether early intervention can prevent the development of diseases.

## Introduction

Many diseases that manifest during early life in dogs are associated with retarded or accelerated growth. Diseases associated with retarded growth include orthopaedic diseases (e.g. nutritional osteodystrophy) [1, 2], congenital endocrine diseases (e.g. hypothyroidism, pituitary dwarfism) [3, 4], alimentary tract disorders (e.g. megaoesophagus, congenital portosystemic shunt (PSS), idiopathic hepatic fibrosis) [5–7], and congenital cardiac diseases e.g. vascular ring anomalies (VRA) [8–12]. Over-nutrition during the growth phase can be associated with developmental musculoskeletal disorders such as hip dysplasia, hypertrophic osteodystrophy (metaphyseal osteopathy), osteochrondritis dissecans, and elbow dysplasia such as fragmented coronoid process and ununited anconeal process [13, 14]. It can also lead to unwanted weight gain and, as in humans [15] and cats [16], this predisposes dogs to obesity later in life [17].

In humans, growth standards, such as those created and promoted by the World Health Organization (WHO) [18, 19] are used to monitor the growth of children, by comparing an individual's pattern of growth with that of a healthy reference population. Such standards can help health professionals to identify individuals with growth disorders more quickly enabling investigations and corrective measures to be implemented [19, 20]. Recently, a series of evidence-based growth standards have been developed for male and female dogs across 5 different size categories and up to 40 kg weight [21]. However, their use in other groups of dogs, in a similar manner to the validation of the WHO growth standards [22, 23], has not been explored. For example, the dogs studied in the original work attended a network of veterinary hospitals across North America and might not be representative of dogs in other locations, with differences in genetics, diet and environmental factors. Further, the possible impact of disease is not known. Therefore, the aim of the current study was to compare growth curves depicted by the standards with patterns of growth in dogs that were either healthy, had abnormal body condition, or had various diseases with the potential to affect growth.

## Materials and methods

### Ethics statement

This study involved analysis of historical data from dogs housed at WALTHAM and Royal Canin pet centres, as well as data analysis of the anonymised records of client-owned dogs attending BANFIELD. Study protocols were reviewed and approved by either the WALTHAM ethical review committee or the Royal Canin Research Ethics Committee.

### Construction of growth standards

The construction of the growth standards used in this study has been described [21]. Briefly, the curves were generated from weight data of healthy dogs in ideal body condition, as recorded in the patient records from BANFIELD^®^ Pet Hospitals, a network of over 900 primary care veterinary hospitals located mainly in the USA. Growth standards were constructed using Generalised Additive Models for Location, Shape and Scale (GAMLSS), as used for the WHO Child Growth Standards [18, 24]. Separate standards were constructed for males and females in 5 different adult weight categories: I (<6.5 kg), II (6.5 kg to <9 kg), III (9 kg to <15 kg), IV (15kg to <30kg) and V (30kg to <40kg).

### Study populations

Data were derived from 2 research populations and BANFIELD^®^ Pet Hospital (BANFIELD) Client Records. **Research Colony Data** came from purebred dogs housed at the WALTHAM Petcare Science Institute (WALTHAM) in the UK and the ROYAL CANIN (RC) Research Centre in France. Dogs were maintained in environmentally enriched housing in accordance with the Animals (Scientific Procedures) Act 1986 Codes of Practice (UK) and the European Directive 2010/63/EU on the protection of animals used for scientific purposes (France) and provided with a structured socialisation programme to meet their mental and physical needs. The studies at WALTHAM and RC were approved by the relevant animal welfare and ethical review bodies. Weekly weights were stored in Microsoft Excel 2013. **Veterinary Client Records Data** were collected from client-owned domestic dogs in BANFIELD^®^ Pet Hospitals between 1994 and 2013, with the data stored in an Oracle 11g patient record database. CS and DW had access to an anonymised copy of the database. Three quarters of the data were from dogs seen from 2003 onwards, due to the expansion of client numbers at BANFIELD^®^ Pet Hospitals over this time.

### Data extraction and eligibility criteria

Each database was searched for dogs under 3 years of age where bodyweight data were available. Dogs were eligible for inclusion in the modelling studies when at least one bodyweight measurement had been recorded between the ages of 0.20 years (~10 weeks) and 2.25 years (2 years 3 months). Extraction of Research Colony Data was conducted using the inbuilt filter function within the electronic spreadsheet (Microsoft Excel). The data of primary interest were bodyweight (between 0.25 and 2 years of age) and contemporaneous age (calculated from the date recorded and the date of birth). Additional data of interest comprised breed, sex, neuter status, and information on body condition. For the Veterinary Client Records Data, the patient records database copy was searched with SQL queries to identify all dogs visiting a BANFIELD^®^ Pet Hospital at least 4 times, over a period of at least 3 months, between the ages of 12 weeks and 2 years. For these dogs, the diagnosis and body condition histories up to 4 years of age were then searched for relevant disease conditions (from diagnoses recorded by the attending veterinarian) to identify subsets of dogs to be used for the validation. For each dog, primary patient information (consisting of patient ID, breed, mixed breed ‘flag’, and date of birth) and details pertaining to the specific visit were gathered. Visit details comprised date, age, weight, body condition (if available) and visit type (e.g. whether for a routine preventative appointment such as vaccination or a non-routine reason). Details of how these data were generated and recorded have previously been described [21]. In addition, the diagnosis or diagnoses recorded by the veterinarian were also reported. In this respect, every time a dog visits a hospital, the attending veterinary must record one or more 'diagnoses' (which can include ‘healthy pet’) by selecting options from a drop-down list in the client record. Standardised criteria were not used for these diagnoses; instead, they were determined by the attending veterinarian based upon their clinical assessment and any diagnostic investigations performed. Given that all variables used for database searching were primary fields, simple search terms were used and no validation of search algorithms was required.

### Final datasets

#### Healthy dogs

Research Colony Data were used to construct three datasets from healthy dogs, which were distinct from each other and also from the growth standards construction dataset [21]. One (**RC Colony Dataset**) was from 12 dogs of 2 breeds based at RC, whilst the second (**WALTHAM Colony Dataset**) comprised data from 430 dogs of 11 breeds based at the WALTHAM centre (Table 1). The third dataset (**Vitamin A Dataset)** was also derived from dogs at the WALTHAM Centre but utilised data from a study to evaluate safety of vitamin A in 48 different dogs of 2 breeds [25]. For dogs in all three groups, body condition was monitored regularly, with corrective action taken where judged to be necessary by colony staff (e.g. reducing food intake when a dog had gained weight too quickly).

**Table 1:** Details of validation datasets.

Dataset	Group	N after cleaning (N before cleaning)			Neutered
		Data	Dogs	Breeds	
RC Colony	N/A	415 (439)	12 (12)	2 pedigree	100%
WALTHAM Colony	N/A	14016 (20375)	368 (430)	11 pedigree	28%
Vitamin A	N/A	2407 (2470)	48 (48)	2 pedigree	65%
Energy Intake					
	Optimal Feeding	223 (240)	6 (6)	1 pedigree	Unknown
	Supplemental feeding	307 (320)	8 (8)	1 pedigree	Unknown
	Restricted feeding	311 (320)	8 (8)	1 pedigree	Unknown
Abnormal Body Condition		1565936 (1729506)	177738 (185029)	228 pedigree & mixed breed	93%
	Obesity	68061 (127939)	8208 (14497)	127	93%
	Overweight	1328512 (1439759)	148804 (158385)	222	95%
	Underweight	145402 (161808)	18247 (19731)	172	76%
Diseases associated with retarded growth		16208 (20860)	1769 (2003)	89	75%
	Dwarfism	211 (372)	29 (43)	17	76%
	Nutritional Osteodystrophy	195 (279)	24 (32)	13	76%
	VRA/PRAA	405 (617)	42 (59)	21	84%
	Portosystemic Shunt	10399 (12428)	1116 (1243)	70	73%
	Hepatic Encephalopathy	1505 (2516)	166 (252)	36	70%
	Megaoesophagus	3493 (4648)	392 (466)	63	85%
Diseases associated with accelerated growth		102316 (155526)	11817 (15565)	152	89%
	Hypertrophic Osteodystrophy	2398 (4270)	262 (441)	38	84%
	Hip Dysplasia (Medical)	78600 (109523)	9087 (11928)	137	89%
	Hip Dysplasia (Surgical)	10782 (19450)	1259 (2095)	83	92%
	Osteochondritis dissecans	7855 (14103)	920 (1434)	79	85%
	Anconeal & Coronoid Process	2681 (5180)	289 (490)	43	88%

RC: ROYAL CANIN; WALTHAM: Waltham Petcare Science Institute; N/A: not applicable; VRA: vascular ring anomaly; PRAA: persistent right aortic arch.

#### Dogs with abnormal body condition

A further two datasets comprised dogs that became either overweight or underweight during growth (Table 1). These datasets were again distinct from each other and from the growth standards construction dataset. The **Energy Intake Dataset** was from an unpublished study where Labrador Retriever puppies from 8 to 52 weeks of age were fed the same diet at different planes of nutrition e.g. 118% (‘supplemental feeding’), 100% (‘optimal feeding’) or 88% (‘restricted feeding’). The **Abnormal Body Condition Dataset** comprised data from dogs that visited a BANFIELD^®^ Pet Hospital at least 4 times, over a period of at least 3 months, between the ages of 12 weeks and 2 years, and which were classified as ‘underweight’, ‘overweight’ or ‘obese’ by 4 years of age.

#### Dogs with diseases associated with altered growth

Data from the Veterinary Records Database were used to identify dogs diagnosed with diseases that might affect the pattern of growth (Table 1). Diagnoses associated with retarded growth were dwarfism, nutritional osteodystrophy, portosystemic shunt, hepatic encephalopathy, megaoesophagus, and persistent right aortic arch/vascular ring anomaly [3–7, 12]. Diagnoses associated with accelerated growth were hypertrophic osteodystrophy, hip dysplasia, osteochondritis dissecans, and the combined category 'anconeal process and coronoid process' (used for ununited anconeal process and fragmented coronoid process) [13, 14]. Again, eligible dogs visited a BANFIELD^®^ Pet Hospital at least 4 times over a period of at least 3 months between the ages of 12 weeks and 2 years and received a diagnosis of one of the selected disease conditions by 4 years of age.

### Data handling and statistical analysis

#### Sample size calculation

A formal sample size calculation was not performed for the study. Instead, for each dataset developed, an attempt was made to include as many dogs as possible that met the eligibility criteria, and to include as many body weights as possible for those dogs.

#### Data cleaning

Data were analysed separately for each of the 5 size categories [21]. Pedigree dogs were assigned to the appropriate category for their breed, whilst mixed breed dogs were categorised by their final adult weight if available, or else excluded. Data were cleaned as before [21]. Box-and-whisker statistics [26] were applied to the weights in each of 40 equal width age categories, and outliers defined by loess lines smoothed across categories fitted to the medians ±150% of the length of the upper and lower whiskers. Gross outliers in individual growth curves were also removed. After data cleaning, individuals with growth trajectories entirely above the 99.6% centile or entirely below the 0.4% centile for their size category were excluded, since this could potentially indicate a misidentified breed.

#### Population-level analysis

Bodyweights were converted to z-scores using the growth standard for the appropriate size category. This converts centile curves to horizontal straight lines, so that abnormal growth appears as a rising or falling line. For the WALTHAM Colony Dataset, the Abnormal Body Condition Dataset (using a random sample of 24,000 data points), and the Diseases Associated with Altered Growth dataset, the z-scores were summarised by fitting Generalised Additive Models for Location, Scale and Shape (GAMLSS) models. GAMLSS is a semi-parametric regression technique where all parameters of the assumed distribution (central tendency, spread, skewness and kurtosis) are modelled as smooth functions of the explanatory variables. Age, size class and their interaction were considered for inclusion in the model (as P-spline smoothed terms with the Schwarz Bayes Criterion as the penalty) using the *t* family distribution implemented within the gamlss package (TF). Model fit was assessed using plots of observed and fitted values, worm plots, normal scores plots and Q-tests as previously described [27]. The Energy Intake and Vitamin A datasets were too small for GAMLSS models to be fitted.

Additional analyses were conducted on the design groups in the Energy Intake dataset, the different body condition score groups in the Veterinary Records Body Condition dataset, and the diagnosis groups in the Diseases Associated with Altered Growth dataset. Mean z-scores were compared amongst different subgroups using a linear mixed model (including dog as a random factor) in conjunction with Tukey post-hoc comparisons.

#### Individual dog-level analysis

For each dog, the number of centile line crossings (i.e. the maximum number of centile lines on the chart crossed in either direction from the starting point) was enumerated and used to quantify extent of deviation from the curves. A subjective comparison between the growth curves and the growth trajectories for dogs from the Research Colony datasets was carried out, taking account of whether individual growth trajectories approximately followed centile lines or whether substantial centile line crossing was seen. The mean differences between the number of centile lines crossed in an upwards direction and the number of centile lines crossed in a downwards direction, for individual dogs, were compared amongst subgroups, using one-way ANOVA with Tukey post-hoc comparisons. Bonferroni adjustment was used to correct for the effects of multiple testing. All analyses were performed with R version 3.1.1 [28], using the gamlss package for the GAMLSS models and the nlme [29] and multcomp [30] packages for the linear mixed models [24].

## Results

### Sample population, data extraction and cleaning

**Fig 1:**
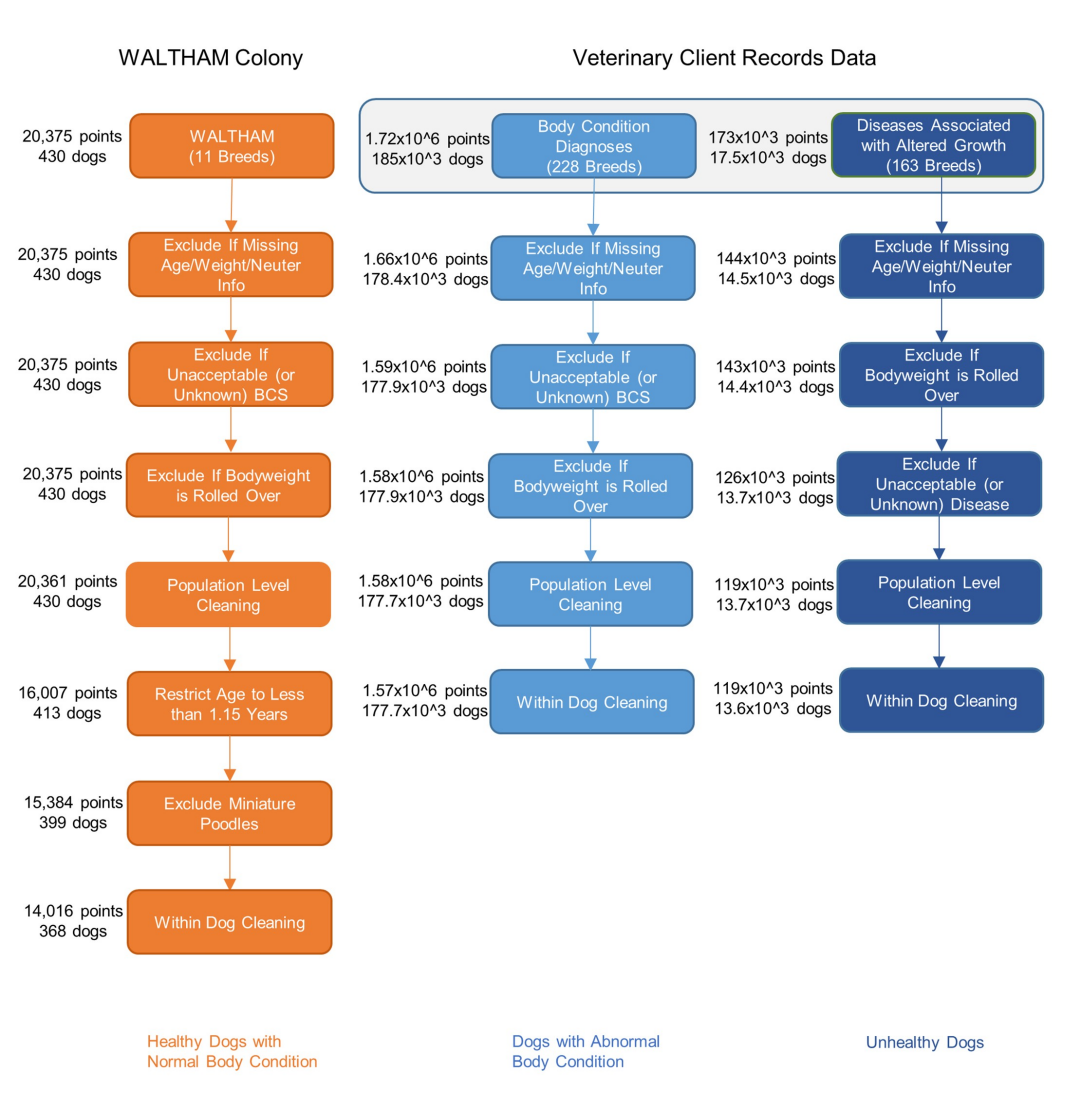
Flow diagram illustrating the data cleaning process. This cleaning process was used for datasets used in the creation of the predicted median growth trajectories. Data are expressed as either thousands (10^3^) or millions (10^6^) as appropriate.

A summary of the dogs and data finally included in each of the studies is given in Fig 1, whilst Table 1 describes features of the validation datasets, including data sizes before and after cleaning. Further details of the breeds of dogs represented in each study are shown in S1 Table. Although not shown in Table 1, neutered dogs in the WALTHAM Colony, RC Colony and Vitamin A datasets underwent the procedure at a median (interquartile range [IQR]) age of 30 (28–54) weeks, 23 (22–24) weeks and 30 (29–31) weeks, respectively. Neutering ages were not universally recorded for the other datasets.

### Individual dog-level analysis

**Fig 2:**
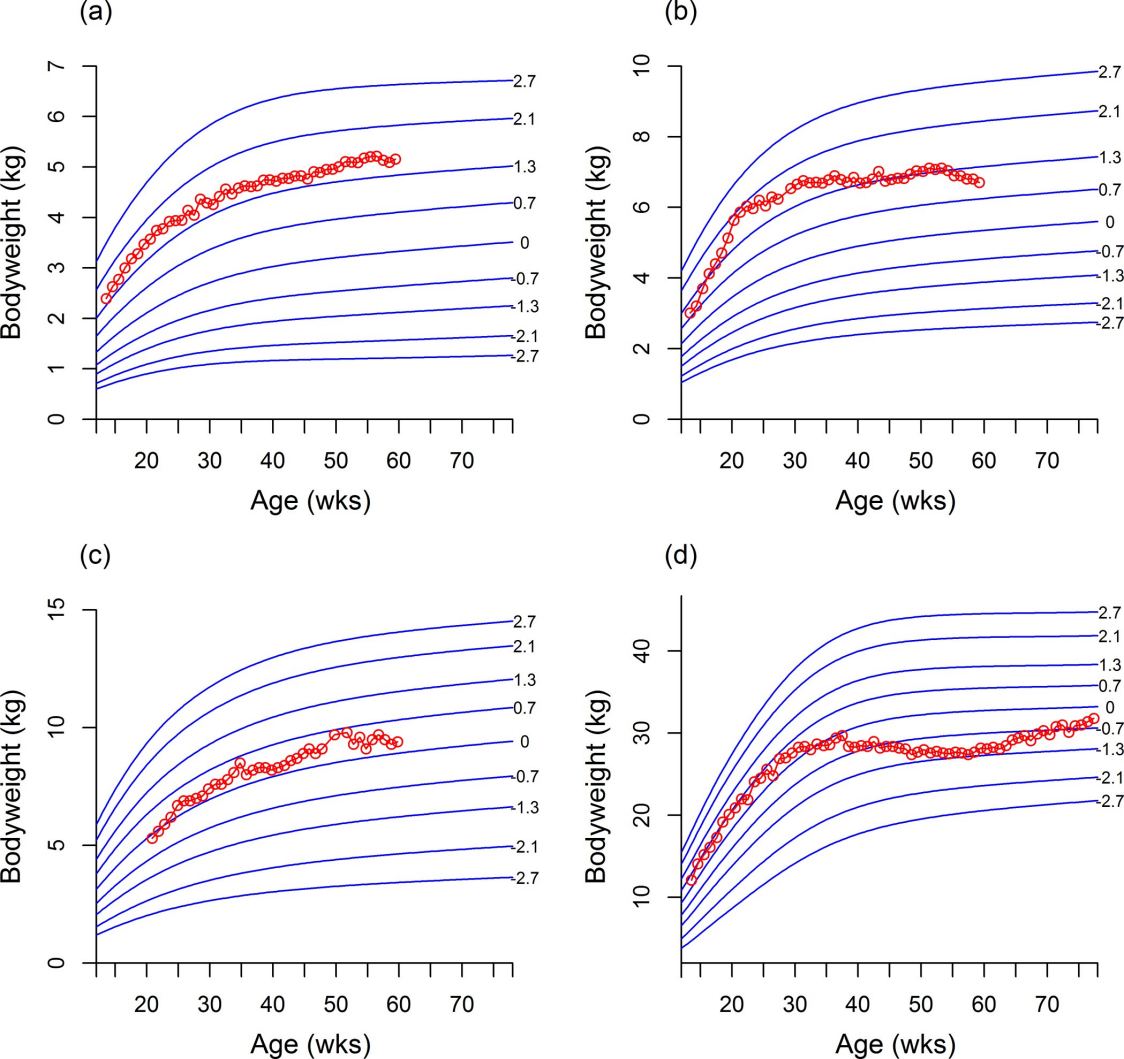
Examples of individual growth trajectories from healthy dogs plotted onto the growth curves. The red open circles represent separate weight measurements for the same dog, whilst the solid blue lines represent the growth curves for the respective breed size category. (a) The growth curve of a male Yorkshire terrier which closely followed the centile lines. (b and c) Growth curves of a female Miniature Schnauzer (b) and a female Cocker Spaniel (c) whose growth pattern followed the centile lines relatively well. (d) Growth curve of a male Labrador retriever whose pattern of growth showed some differences from the centile lines, as indicated by some centile line crossing.

**Fig 3:**
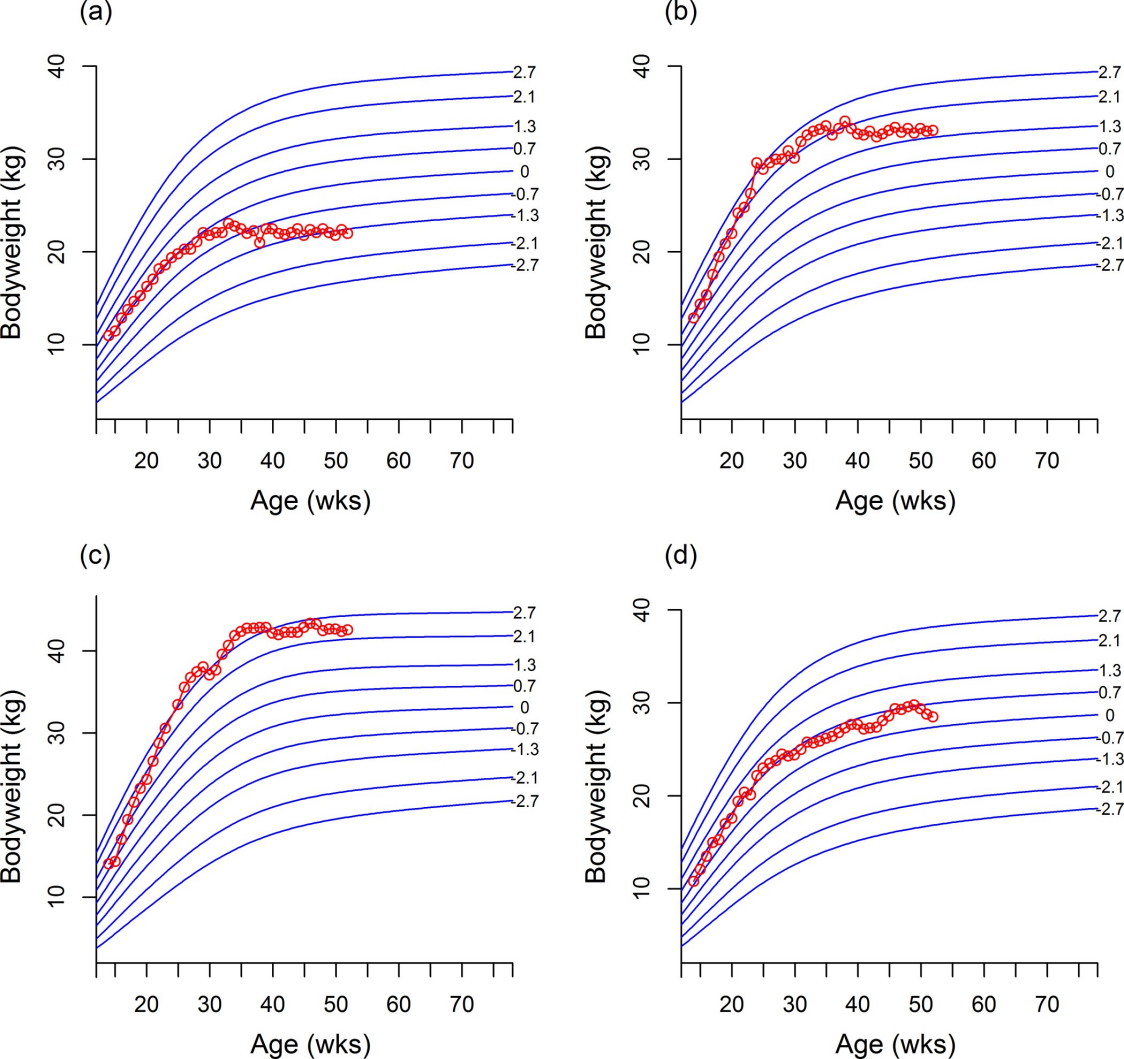
Examples of individual growth trajectories from dogs on restricted and supplemental feeding diets. The red open circles represent weights, whilst the solid blue lines represent the growth centiles for the respective breed size category. (a) A female Labrador Retriever on a restricted diet crossing centile lines on a downwards trajectory. (b) Growth curves from a female Labrador Retriever on a supplemental diet crossing multiple centile lines and (c) a male Labrador Retriever on a supplemental diet crossing centile lines on an upwards trajectory. (d) Growth curve from a female Labrador Retriever on a supplemental diet showing no centile line crossing.

Table 2 summarises the number of centile line crossings in each of the validation datasets. Centile line crossing was rare for the healthy dogs (WALTHAM and RC) with <4% crossing >2 centile lines, but more frequent among the unhealthy dogs. In the healthy dog datasets, 42% from WALTHAM and 8% from RC crossed >1 centile line. Figs 2 and 3 show examples of dogs whose growth trajectory either tracked well or less well the centile lines of the standards.

**Table 2:** Centile line crossings (at the most differential point away from an individual’s starting centile) in the validation datasets used in the study.

Type	Dataset	Group	Total centile line crossings				Upward centile lins crossings				Downward centile line crossings			
			0	1	2	>2	0	1	2	>2	0	1	2	>2
Healthy	WALTHAM/RC colonies	WALTHAM Population	37 (10%)	174 (48%)	133 (37%)	16 (4%)	16 (4%)	66 (18%)	53 (15%)	5 (1%)	21 (6%)	108 (30%)	80 (22%)	11 (3%)
		RC Population	0 (0%)	11 (92%)	1 (8%)	0 (0%)	0 (0%)	2 (17%)	0 (0%)	0 (0%)	0 (0%)	9 (75%)	1 (8%)	0 (0%)
	Vitamin A	N/A	1 (2%)	19 (40%)	20 (42%)	8 (17%)	0 (0%)	12 (25%)	7 (15%)	1 (2%)	1 (2%)	7 (15%)	13 (27%)	7 (15%)
	Energy intake study	Optimal feeding	0 (0%)	2 (33%)	2 (33%)	2 (33%)	0 (0%)	1 (17%)	0 (0%)	0 (0%)	0 (0%)	1 (17%)	2 (33%)	2 (33%)
Abnormal body condition	Energy intake study	Supplemental feeding	0 (0%)	1 (12%)	3 (38%)	4 (50%)	0 (0%)	1 (12%)	1 (12%)	1 (12%)	0 (0%)	0 (0%)	2 (25%)	3 (38%)
		Restricted feeding	0 (0%)	1 (12%)	6 (75%)	1 (12%)	0 (0%)	0 (0%)	0 (0%)	0 (0%)	0 (0%)	1 (12%)	6 (100%)	1 (12%)
	Body condition veterinary records	Overweight	13199 (9%)	45916 (31%)	45578 (31%)	44111 (30%)	8394 (6%)	29702 (20%)	35067 (24%)	39293 (26%)	4805 (3%)	16214 (11%)	10511 (7%)	4818 (3%)
		Obesity	644 (8%)	1,946 (24%)	2,327 (28%)	3,291 (40%)	438 (5%)	1358 (17%)	1879 (23%)	3010 (37%)	206 (3%)	588 (7%)	448 (5%)	281 (3%)
		Underweight	2,343 (13%)	6,949 (38%)	5,499 (30%)	3,456 (19%)	1033 (6%)	2865 (16%)	2258 (12%)	1691 (9%)	1310 (7%)	4084 (22%)	3241 (18%)	1765 (10%)
Unhealthy	BANFIELD^®^ retarded growth conditions	Dwarfism	8 (28%)	9 (31%)	11 (38%)	1 (3%)	2 (7%)	4 (14%)	4 (14%)	1 (3%)	6 (21%)	5 (17%)	7 (24%)	0 (0%)
		Nutritional Osteodystrophy	7 (29%)	7 (29%)	4 (17%)	6 (25%)	4 (17%)	4 (17%)	2 (8%)	5 (21%)	3 (13%)	3 (13%)	2 (8%)	1 (4%)
		PRAA/VRA	3 (7%)	21 (50%)	12 (29%)	6 (14%)	1 (2%)	11 (26%)	3 (7%)	4 (10%)	2 (5%)	10 (24%)	9 (21%)	2 (5%)
		Portosystemic Shunt	158 (14%)	541 (48%)	313 (28%)	104 (9%)	62 (6%)	236 (21%)	161 (14%)	63 (6%)	96 (9%)	305 (27%)	152 (14%)	41 (4%)
		Hepatic Encephalopathy	19 (11%)	85 (51%)	38 (23%)	24 (14%)	5 (3%)	41 (25%)	19 (11%)	9 (5%)	14 (8%)	44 (27%)	19 (11%)	15 (9%)
		Megaoesophagus	52 (13%)	155 (40%)	124 (32%)	61 (16%)	20 (5%)	70 (18%)	65 (17%)	35 (9%)	32 (8%)	85 (22%)	59 (15%)	26 (7%)
	BANFIELD^®^ accelerated growth conditions	Hypertrophic osteodystrophy	23 (9%)	94 (36%)	80 (31%)	65 (25%)	11 (4%)	43 (16%)	45 (17%)	44 (17%)	12 (5%)	51 (19%)	35 (13%)	21 (8%)
		Hip Dysplasia, Medical	932 (10%)	3,260 (36%)	2,700 (30%)	2,195 (24%)	444 (5%)	1768 (20%)	1628 (18%)	1668 (18%)	488 (5%)	1492 (16%)	1072 (12%)	527 (6%)
		Hip Dysplasia, Surgical	130 (10%)	450 (36%)	392 (31%)	287 (23%)	53 (4%)	250 (20%)	231 (18%)	220 (17%)	77 (6%)	200 (16%)	161 (13%)	67 (5%)
		Osteochondritis dissecans	94 (10%)	315 (34%)	285 (31%)	226 (25%)	41 (4%)	171 (19%)	170 (18%)	170 (18%)	53 (6%)	144 (16%)	115 (13%)	56 (6%)
		Anconeal and coronoid process	23 (8%)	109 (38%)	89 (31%)	68 (24%)	13 (4%)	61 (21%)	53 (18%)	51 (18%)	10 (3%)	48 (17%)	36 (12%)	17 (6%)

RC: ROYAL CANIN; WALTHAM: Waltham Petcare Science Institute; N/A: not applicable; VRA: vascular ring anomaly; PRAA: persistent right aortic arch.

### Population-level analysis

#### Predicted median growth trajectories in healthy dogs

**Fig 4:**
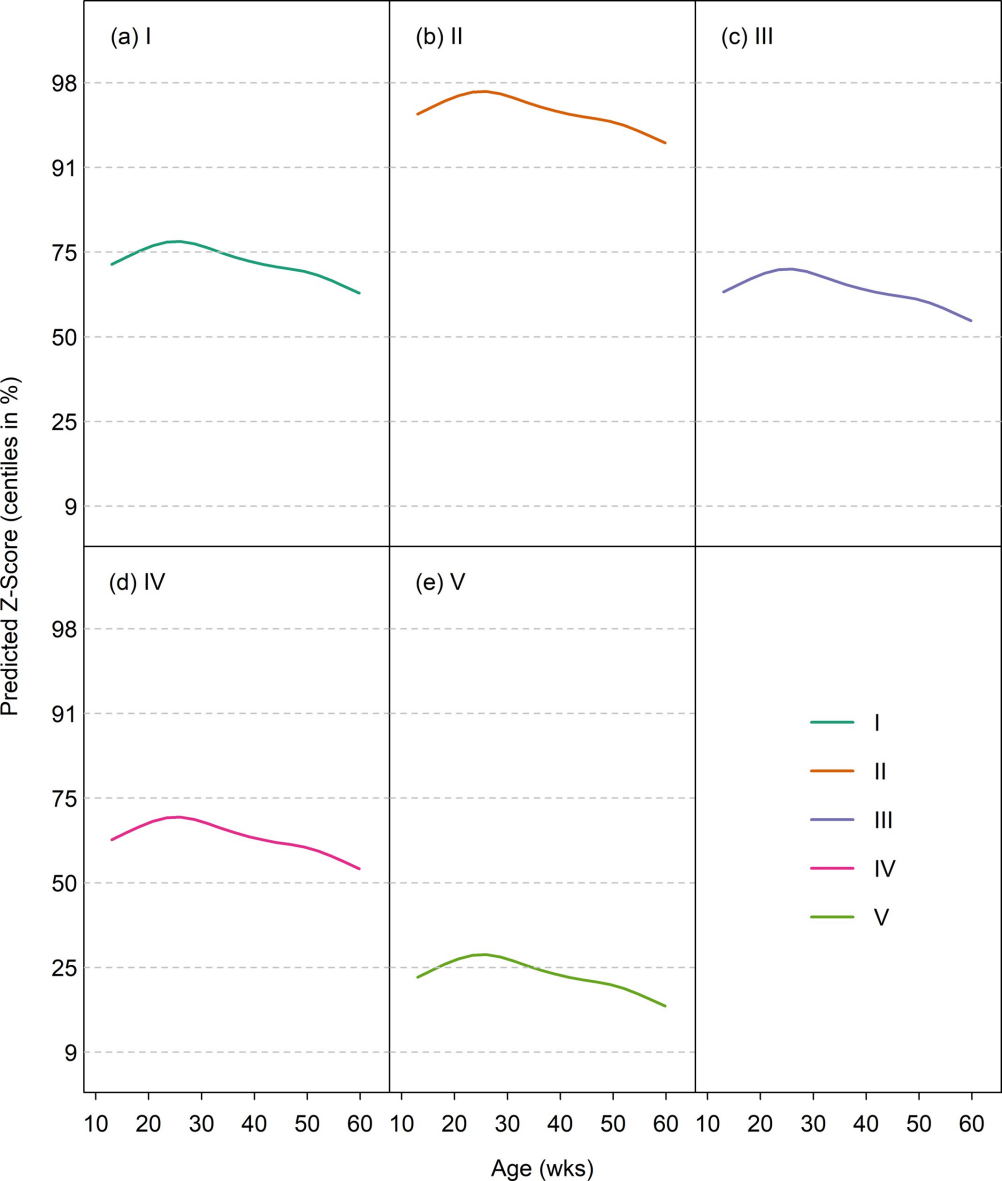
Median weight by age (on the centile scale) for the WALTHAM Research Colony dataset in 5 size classes. On each graph, the median for the validation dataset is depicted by the solid line, whilst the growth standard centiles are indicated by dotted horizontal lines.

The GAMLSS model fitted to weight z-score for the WALTHAM Colony dataset included size class and P-spline smoothed age terms. Interaction terms were tested but did not improve the model. Age was truncated at 1.15 years as there were few older data. Fig 4 shows for each size category the predicted median curve superimposed on the growth standard centiles (on the z-score scale). The fitted curves rose initially and then fell, peaking between 20 and 30 weeks. They lay between the median and 75^th^ centile for classes I, III and IV, but above the 91^st^ centile for class II and below the 25^th^ centile for class V. The fact that the fitted curves did not lie on the 50^th^ centile was not unexpected since the colony contained a limited number of dog breeds within each size class compared with the population used to create the growth standards (S1 Table), [21]. For example, miniature schnauzers and Labrador retrievers were the only breeds represented in size category II and V, respectively; miniature schnauzers are one of the heaviest breeds in size category II, whilst Labrador retrievers are one of the lighter breeds in size category V.

#### Predicted growth trajectories in dogs with abnormal body condition

**Fig 5:**
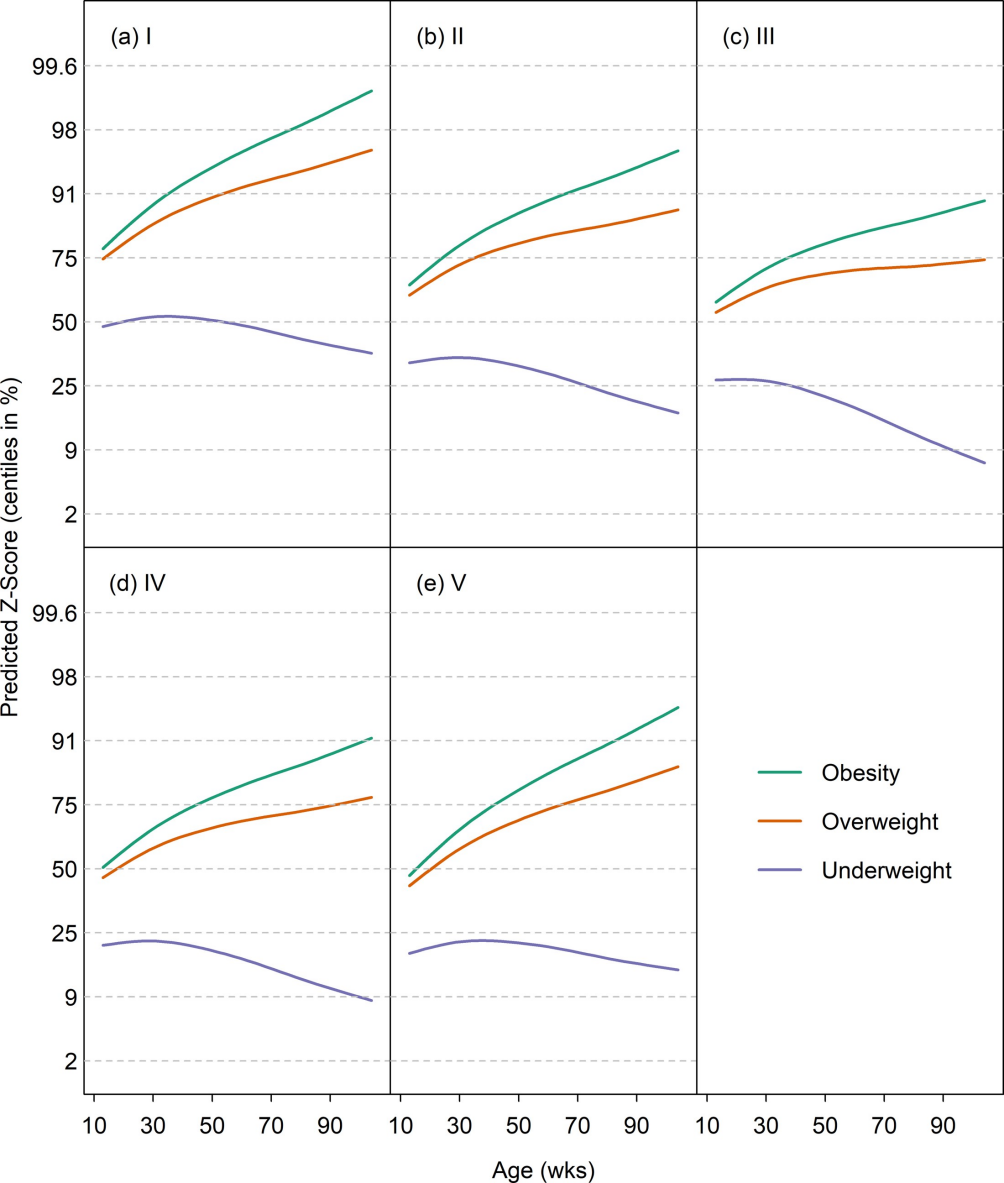
Median weight curves by age (on the centile scale) for obese, overweight and underweight dogs from the Body Condition Veterinary Records dataset in 5 size classes. The trajectories for obese, overweight and underweight dogs are depicted by solid light green, tawny, and light blue lines, respectively, whilst the growth standard centiles are indicated by dotted horizontal lines. For obese and overweight dogs the centiles cross upwards, while for underweight dogs they cross downwards.

The GAMLSS model for the Abnormal Body Condition Dataset contained the terms visit age, diagnosis age, diagnosis group (i.e. underweight, overweight or obese) and size class. Fig 5 shows the predicted median curves (on the z-score scale) for the three diagnosis groups for each size category. The predicted means were initially near the 50th centile, but crossed centile lines upwards in the overweight and obesity diagnosis groups, and downwards in the underweight group. Z-scores were also compared amongst the various groups in the Energy Intake Dataset. There were significant differences in median z-score between all groups (supplemental feeding vs. optimally-fed, difference in z-score 1.36, adjusted 95%-CI 1.18 to 1.54; supplemental feeding vs. restricted feeding difference in z-score 2.0, adjusted 95%-CI 1.83 to 2.16; optimally-fed vs. restricted feeding difference in z-score 0.64, adjusted 95%-CI 0.46 to 0.82).

#### Predicted growth trajectories for diseases associated with retarded and accelerated growth

**Fig 6:**
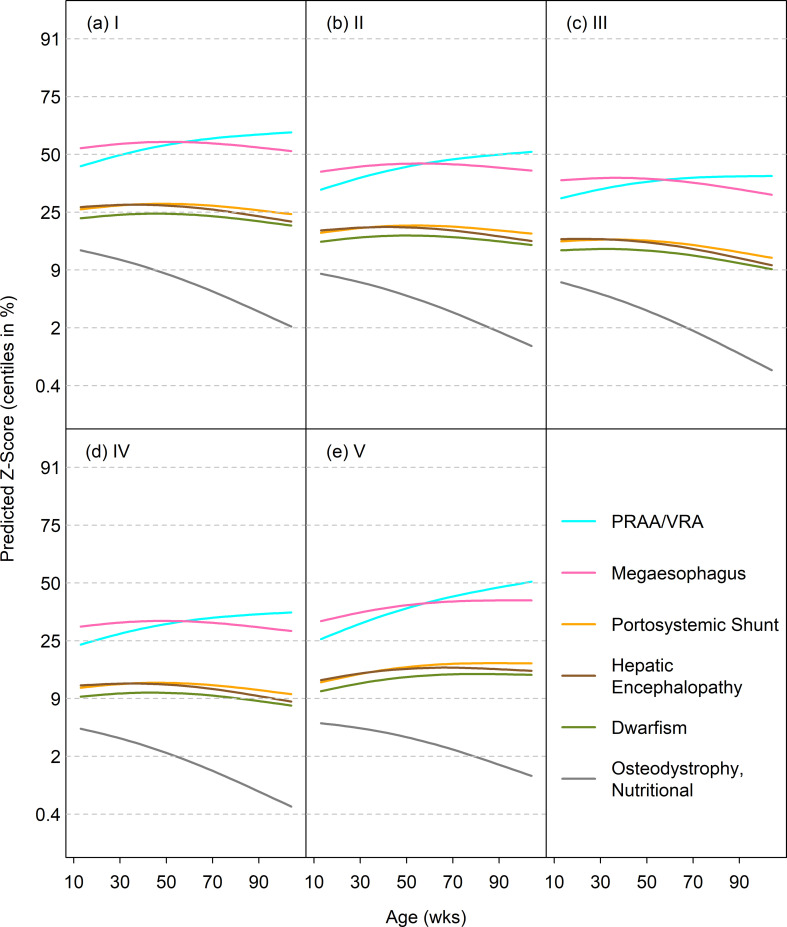
Median weight curves (on the centile scale) for diseases associated with retarded growth in 5 size classes. Diseases are persistent right aortic arch/vascular ring anomaly (blue), megaoesophagus (pink), portosystemic shunt (yellow), hepatic encephalopathy (brown), dwarfism (green) and 'osteodystrophy, nutritional' (grey).

**Fig 7:**
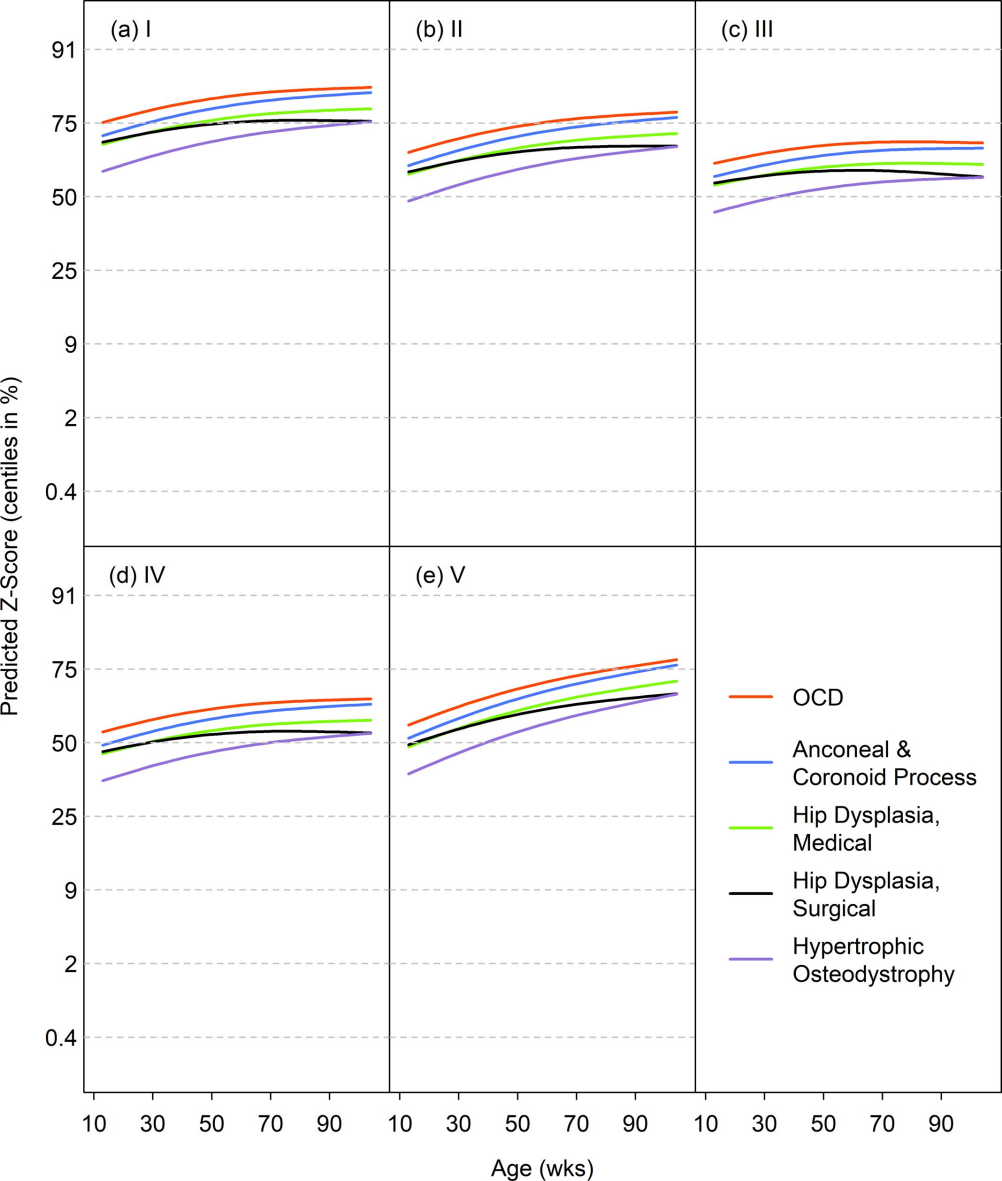
Median weight curves (on the centile scale) for diseases associated with accelerated growth in 5 size classes. Diseases are OCD (red), ‘anconeal & coronoid process’ (blue), ‘hip dysplasia, medical’ (green), ‘hip dysplasia, surgical’ (black) and hypertrophic osteodystrophy (purple).

The disease-specific GAMLSS models included terms for age, diagnosis age, diagnosis group and size class. For most diseases associated with retarded growth, the predicted median curves by age followed either the 25^th^ or 9^th^ centile, indicating the dogs were relatively light (Fig 6). The curves for megaoesophagus and PRAA/VRA were somewhat greater, between the 25^th^ and 50^th^ centiles. The one disease to show a clear falling growth pattern was nutritional osteodystrophy, starting near the 9th centile and falling steeply. Fig 7 shows the predicted median curves by age for each size class for diseases associated with accelerated growth. Most of the curves started between the 50^th^ and 75^th^ growth standard centiles, and rose modestly with age. Therefore, the curves were on average one channel width higher for accelerated as compared to retarded growth.

## Discussion

The findings of the current study have extended the original work in creating canine growth reference standards, by comparing growth patterns in other groups of healthy dogs and in dogs that developed abnormal body condition and a range of disorders associated with growth depicted by the curves of the standard. Overall, patterns of growth in the healthy dogs assessed were similar, and it was unusual to see the crossing of two or more centile lines in the same direction; in the WALTHAM cohort, 36% did cross up to two centile lines, but only 5% of dogs crossed more than 2 centile lines. In contrast, growth patterns of dogs with an abnormal body condition and those diagnosed with disorders affecting growth rate frequently differed from that depicted by the growth standards. For example, 83% of dogs that had become overweight by early adulthood crossed at least two centile lines in an upwards direction, whilst the same was true of 67% of dogs diagnosed with diseases associated with accelerated growth. Further, crossing centile lines in a downwards direction was more likely, though less marked in dogs that had become underweight by early adulthood (where 80% crossed tat least two centile lines in a downwards direction) or were diagnosed with diseases associated with retarded growth (where 37% crossed at least two centile lines in a downwards direction). Taken together, these results suggest that the growth standards assessed in the current study could form the basis of a clinical tool for monitoring healthy growth in dogs.

Although the use of the growth standard has potential as a tool to alert veterinary professionals to possible abnormalities in growth, it is not a diagnostic test. In this respect, identifying centile line crossing increases the chance that a growth disturbance is present, but does not guarantee it because some healthy dogs can display similar patterns of growth. Furthermore, some dogs that became overweight or developed diseases associated with growth crossed <2 centile lines, most notably for conditions leading to retarded growth. Such limitations are not unexpected because the same caveats exist for human growth standards, whereby centile line crossing increases the odds of a child becoming obese but this does not invariably occur [31]. Therefore, such growth standards are best used as an aid to support decision making by veterinary professionals, and in helping conversations with owners regarding nutrition and husbandry. For example, where centile line crossing is identified, it could prompt a veterinary professional to review the nutrition and husbandry of the dog and consider the need for diagnostic investigations. This could help to identify problems earlier, such that corrective measures can then be implemented, or to enable veterinary professionals to implement monitoring strategies for individuals considered to be at increased risk.

Our first objective was to compare the pattern of growth depicted in the growth standards with growth in separate populations of healthy dogs. For this, bodyweight data from two colonies of healthy dogs were used. Individual analysis demonstrated that centile line crossing was relatively common, with most dogs crossing at least one centile line. However, it was less common for dogs to cross two centile lines, and rare for more than two centile lines to be crossed. Further, dogs could cross centile lines in either direction. The results of population-level analysis of data from the healthy dog populations support these findings since growth trajectory (on the z-score scale) was approximately horizontal in all size categories, with deviations being small and well within the distance between successive centile lines. Taken together, these results suggest that, whilst there is some individual variation, the growth depicted in the standards is a reasonable representation of growth in domesticated dogs. This is notable because the populations used for validation were from different geographical locations and had different husbandry. Of course, the main limitation of the populations chosen was the fact that dogs from research colonies were used, rather than data from pet dogs, because accurate historical data were readily available for analysis. Ideally, further studies using different populations of healthy pet dogs should be considered in order to provide additional information on the validity of these standards to the wider pet population.

Two further observations regarding the analysis of healthy dog growth are of note. Firstly, the overall trajectory of the median z-score was similar amongst size categories; with a slight initial upwards inclination followed by a subsequent downwards inflection. This pattern is consistent with the trajectory in many individual dogs. The reason for this pattern of change is not clear, but the fact that the change was similar across size categories could suggest differences in either environment or husbandry between dogs in the validation set and the population used to create the growth standards. It is not uncommon to identify such minor differences in patterns of healthy growth between populations; indeed, when studies were conducted to determine the suitability of the WHO growth standards for use in the UK, differences were observed in growth patterns of the populations used to validate them [22]. In this respect, British children were born heavy for age, with subsequent 'catch-down' growth followed by growth that was more rapid than depicted by the growth standards. The second observation of note was that the median predicted z-score did not lie on the 50th centile in any of the categories, instead ranging from a position close to the 98th centile (size category II) to the 25th centile (size category V). As mentioned above, the most likely reason for this was the fact that the breeds represented in the research colony were limited compared with the population used to create the standards. However, it could instead be related to differences in stature of the dogs in the breeds in this population (which was from the UK) compared with the stature of the dogs of the equivalent breeds in the references (which was from the USA). In this respect, selective breeding might have led to a divergence in the gene pools of each breed in the different countries. Further work would be required to determine the reasons for the trends seen within these populations.

The second objective was to assess differences in the pattern of growth amongst dogs on different planes of nutrition. This aspect of the study utilised data from a study in which groups of dogs were fed differing energy intakes during growth. Dogs that received supplemental feeding grew faster and had a significantly greater median z-score than the two other groups, and half of them crossed more than two centile lines, suggesting that over-nutrition has a marked effect on weight gain. In contrast, there was no significant difference in median z-score between dogs fed optimally and those fed a restricted energy intake. This implies that individual dogs in the supplemental feeding group, on average, tracked higher centile lines than individuals in the optimally fed or restricted groups, although with no evidence that the differences were increasing with age. This might either be because changes in growth between the groups had already stabilised by 12 weeks of age, or might instead reflect the fact that the dataset was small and was thus underpowered for this analysis. A final explanation was that, given the ethical considerations of this study, the degree of energy restriction that was used was modest, and minimised the effects observed.

The third objective of this study was to assess patterns of growth in groups of dogs that developed an abnormal body condition within the first three years of life. This included dogs diagnosed by the attending veterinarian as underweight, overweight and obese. For overweight and obese dogs, upward centile line crossing was common, with approximately 61% and 68% of overweight and obese dogs, respectively, crossing at least two centile lines. This suggests that dogs in these two categories had a faster rate of weight gain, on average, than the growth curves would suggest. Conversely, the median z-score trajectories for the underweight diagnosis group sloped downwards, indicating a slower growth rate, although the trajectory was less steep especially in size categories I and V. Most striking was the fact that, for each of these categories (obese, overweight and underweight), the predicted trend was observable from 26 weeks, if not before. Although such summary trends may be more easily detectable by eye than patterns in individuals’ measurements, this demonstrates that the growth standards have, at the least, a potential for early identification of an abnormal growth pattern by veterinary professionals, which in the future could enable corrective measures to be implemented before there is a clinical problem. For example, food intake could be reduced in dogs identified as growing more quickly than expected, whilst food intake could be increased, and/or diagnostic investigations considered in dogs identified as growing too slowly. Of course, as is the case with the WHO growth standards for children [32], clear guidance would be required for veterinary professionals as to optimal monitoring strategies, intervention points, and the type of action needed if abnormal growth patterns are identified. It should also be noted that centile line crossing was not a perfect predictor of future weight status. Whilst centile line crossing was usually in an upwards direction, some dogs could cross centile lines downwards yet were still diagnosed as overweight or obese by three years of age. Although the exact reason for such a finding is not clear, one possible reason is if the dogs gained excessive weight after completion of the growth phase. Therefore, this emphasises the fact that growth standard monitoring is not perfect and should be considered alongside other methods of assessing weight status such as body condition scoring.

The final objective was to determine the influence of various diseases previously associated with either retarded or accelerated growth. These results should be interpreted with caution because the number of dogs included in many categories was small, and because diagnosis categories were assigned by the attending veterinarian, meaning that criteria used were not standardised. Therefore, the broad trends are arguably more important than specific details. Six of the diagnosis categories were chosen because they have previously been associated with retarded growth [3, 6, 7, 12], and the growth trajectory was relatively flat for the majority suggesting no marked deviation from the growth depicted by the growth standards. However, since the predicted median z-score usually started below the 50th centile, which denotes a median ‘normal’ dog on this scale, dogs might have already been small-for-age at 12 weeks. This either suggests that these conditions are associated with lower birth weight puppies (either because the individual puppies are small-for-breed or because the conditions are more associated with the smaller breeds in each size class), or that the influence of the disease condition commenced at a very young age. Therefore, the predicted growth trajectories might only be demonstrating the tail end of an already established pattern of failure to thrive. A further explanation for the flat trajectory could be the influence of corrective therapy after diagnosis, for instance surgical correction in cases of PSS and VRA, and postural feeding in dogs with megaoesophagus. In contrast to this flat growth trajectory, marked growth retardation was seen in dogs with nutritional osteodystrophy (nutritional secondary hyperparathyroidism) with predicted z-scores at 12-weeks of age below the 9th centile, and a subsequent downwards growth trajectory with 30% of dogs crossing two or more centile lines. The reason why changes were particularly noticeable for this condition is not known, but findings should be interpreted cautiously because it was a rare disease category and the small number might not be fully representative.

The study had some limitations. First, the study utilised data from a variety of populations of differing sizes, with data sources selected because they were available to the authors, rather than having been prospectively collected for validation of the growth standards. Most notable were the data used from healthy dogs, which were from small numbers of dogs in two research colonies. Therefore, the findings might not be generalisable to groups of pet dogs in other geographical locations. Arguably, datasets used to assess abnormal body condition and diseases affecting growth were much larger and were more representative since they were derived from a population of pet dogs attending veterinary hospitals across North America. That said, the same source was used to obtain the original dataset used to create the growth standards, and this again means that the findings are not necessarily generalisable to dogs from other geographical locations. A second limitation is the fact that data (both for this study and for the generation of the standards) were collected retrospectively from many locations, by many people, over a period of over 20 years. This raises the possibility that the older data might not be representative of dogs living today, not least given the changes in the types of foods that are popularly fed as well as other environmental factors. In addition, the criteria used to assign dogs to different diagnosis for many of the disease diagnoses was not standardised. Many changes would have been expected in veterinary practice protocols, expertise, and technology over this timeframe. Thus, there may have been inaccuracies in diagnosis, meaning that the groups studied were not representative of the conditions that they were meant to represent, with greatest concern being for the rare conditions with small group sizes. Therefore, further validation studies are now recommended to assess patterns of growth using prospectively collected data from dogs in different populations.

A final limitation (which is more a limitation of growth monitoring) arising from the use of retrospective data is that whilst growth patterns differing from that depicted by the growth standards have been identified for many of the conditions studied, causality cannot be assumed. Therefore, identifying an abnormal growth pattern and then correcting it might not prevent the condition from occurring. Future prospective studies would be required to determine whether growth monitoring can have a beneficial effect on disease prevention.

## Conclusions

In the current study, the growth patterns of different healthy and unhealthy groups of dog have been compared with recently-created growth standards for monitoring bodyweight in dogs. Crossing centile lines in an upwards direction is associated with dogs that become overweight by early adulthood or have developmental orthopaedic disorders, especially if two or more centile lines are crossed. Similarly, crossing centile lines in a downwards direction is associated with dogs that become underweight by early adulthood or have diseases associated with retarded growth. Prospective studies are now required to confirm these findings and to determine whether early intervention can prevent the development of diseases.

## Supporting information

S1 Table Breed details for three colony trial datasets. (DOCX)
