# Supplementary material for: Comparison of growth patterns in healthy dogs and dogs in abnormal body condition using growth standards
Source: PLoS One. 2020 Sep 23;15(9):e0238521. doi: 10.1371/journal.pone.0238521 (PMC7510995; doi:10.1371/journal.pone.0238521)
Supplement: S1 Table — (DOCX) [file pone.0238521.s001.docx]

**S1 Table.**  **Breed details for three colony trial datasets.**

| **Dataset** | **Breed** | **Size Class** | **Data** | **Dogs** |
| --- | --- | --- | --- | --- |
| WALTHAM population | Papillon | I | 174 | 5 |
|  | Yorkshire Terrier | I | 1,359 | 40 |
|  | Miniature Schnauzer | II | 4,082 | 89 |
|  | Standard Dachshund | II | 75 | 2 |
|  | Cocker Spaniel | III | 1,495 | 39 |
|  | Standard Schnauzer | III | 21 | 2 |
|  | Clumber Spaniel | IV | 37 | 2 |
|  | Field Spaniel | IV | 15 | 1 |
|  | Greyhound | IV | 7 | 7 |
|  | Labrador Retriever | V | 6,751 | 181 |
| Royal Canin Population | Miniature Schnauzer | II | 131 | 4 |
|  | West Highland White Terrier | II | 284 | 8 |
| Vitamin A | Labrador Retriever | V | 1,491 | 23 |
|  | Miniature Schnauzer | II | 916 | 25 |
| Energy intake study |  |  |  |  |
| Optimal feeding | Labrador Retriever | V | 223 | 6 |
| Supplemental feeding | Labrador Retriever | V | 307 | 8 |
| Restricted feeding | Labrador Retriever | V | 311 | 8 |
